# Description of a new species of *Sternocoelis* from Morocco with proposal of the *Sternocoelis marseulii* species group (Coleoptera, Histeridae)

**DOI:** 10.3897/zookeys.181.2953

**Published:** 2012-04-06

**Authors:** Tomáš Lackner, Peter Hlaváč

**Affiliations:** 1 Czech University of Life Sciences, Faculty of Forestry and Wood Sciences, Department of Forest Protection and Game Management, Kamýcká 1176, CZ-165 21 Praha 6 – Suchodol, Czech Republic; 2 Na Doline 14, 044 14, Košice, Slovakia

**Keywords:** Coleoptera, Histeridae, Haeteriinae, *Sternocoelis*, new species, Morocco, Spain, Algeria

## Abstract

The*Sternocoelis marseulii* species group is proposed based on antennal and prosternal characters. Five species are included in the group:*Sternocoelis marseulii* (Brisout de Barneville, 1866)(Spain), *Sternocoelis viaticus* Lewis, 1892 (Algeria), *Sternocoelis vaucheri* Lewis, 1896 (Morocco), *Sternocoelis berberus* Lackner & Yélamos, 2001 (Morocco)and *Sternocoelis yelamosi* **sp. n.** (Morocco). The external morphology of *Sternocoelis yelamosi* **sp. n.** is described and illustrated, the illustrations of genitalia of all species of the group (except for *Sternocoelis vaucheri*) are provided and a key to the species of the group is given.

## Introduction

The genus *Sternocoelis* Lewis, 1888 is a small genus of myrmecophilous histerids with 27 described species distributed in the Mediterranean area with most species described from Morocco and Algeria (Yélamos 1995, Mazur 1997). The genus has been recently revised by Yélamos (1995). Since then only one further species has been described, from the Moroccan High Atlas (Lackner and Yélamos 2001). Recently, mainly Slovak and Czech coleopterists have carried out numerous expeditions with focus on the myrmecophilous beetles of Morocco. The newly described species from the Moroccan Middle Atlas results from one such expedition and shows that our knowledge of the Moroccan fauna of *Sternocoelis* is still incomplete.

## Material and methods

Beetles, after being removed from original cards, were side-mounted on triangular points and examined under Nikon 102 binocular microscope with diffuse light. Male genitalia were first macerated in 10% KOH solution for about 15 minutes, cleared in 80% alcohol and macerated in lactic acid with fuchsine heated up to 60°C for another two hours. After that, they were treated with aceto-salycilate heated up to 60°C for 15 minutes and cleared in xylene. They were subsequently examined in α-terpineol in a small dish. Digital photographs were taken by a Nikon 4500 Coolpix camera and edited in Adobe Photoshop CS3. Based on the photographs, observing the actual genitalia, pencil art was drawn; pen art followed, re-tracing the pencil art and making minor corrections. SEM photographs were taken by Hitachi S-2250N camera.

The following acronyms of museums and private collections are used throughout the text:

BMNH The Natural History Museum, London, United Kingdom (R. Booth);

CTLA Tomáš Lackner collection, Leiden, The Netherlands;

CTYB Tomás Yélamos collection, Barcelona, Spain;

MNHN Muséum National d’Histoire Naturelle, Paris, France (A. Taghavian).

### Abbreviations

Abbreviations of morphological measurements follow Ôhara (1994) and are used throughout the text as follows:

APW width between anterior angles of pronotum

EL length of elytron along elytral suture

EW maximum width between outer margins of elytra

PEL length between anterior angles of pronotum and apices of elytra

PPW width between posterior angles of pronotum.

Separate lines of the same label are marked by slash (/); separate labels are marked by double slash (//). Morphological conventions and terminology and methods of illustration preparation follow Lackner (2010).

## Taxonomy

### Sternocoelis marseulii species group

All members of the group can easily be distinguished from all other *Sternocoelis* by the combination of the following character states:

1. prosternal lobe at the same level or slightly below the level of prosternal keel;

2. prosternal lobe not divided medially and without deep emargination;

3. prosternal keel with carinal prosternal striae distinct and (almost) joined anteriorly;

4. elytra with first dorsal elytral stria complete;

5. antennal scape with a ‘hook’ (except for *Sternocoelis viaticus*).

Yélamos (1995), in his revision of the genus placed species *Sternocoelis marseulii* (Brisout de Barneville, 1866), *Sternocoelis vaucheri* Lewis, 1896 and *Sternocoelis viaticus* Lewis, 1892 in a small clade closest to the out-group (*Haeterius ferrugineus* (Olivier, 1789)). *Sternocoelis berberus* Lackner & Yélamos, 2001 and *Sternocoelis yelamosi* (described here) share with the three afore-mentioned species identical character states, so we believe that these two species also belong in this plesiotypic clade, which we define as ‘*Sternocoelis marseulii* species group’. Although the monophyly of this group is highly likely, it requires testing by a phylogenetic analysis in the future. A phylogenetic analysis of *Sternocoelis* would be, however, outside of the scope of this paper. We are aware of the fact that proposing new taxonomic structure without a real phylogenetic analysis to support it should not be a standard measure. In the present work we therefore designate this species-group mostly on pragmatic grounds as an informal taxonomic unit that should serve as a pointer for the future studies of this difficult genus. We believe that male terminalia should be examined in all extant species with the special focus on the male terminalia and spiculum gastrale in particular (see Discussion). All five species included in the group are externally rather similar and, for secure identification, the examination of the male terminalia, especially spiculum gastrale, is necessary.

### Key to the species of the Sternocoelis marseulii group:

**Table:** 

1 (8)	Antennal scape with a ‘hook’ (Fig. 20)
2 (3)	Prosternum, especially apically, weakly punctate (Fig. 4)	*Sternocoelis yelamosi* sp. n. (Morocco: Jebel Tazzeka)
3 (2)	Prosternum apically very coarsely and densely punctate, rugose (Fig. 17)
4 (5)	Pronotal sides strongly explanate (see Lackner and Yélamos 2001: fig. 1), well-separated from disc, body size 1.40–1.60 mm	*Sternocoelis berberus* Lackner & Yélamos, 2001 (Morocco: High Atlas)
5 (4)	Pronotal sides weakly explanate, not well separated from the disc (Fig. 30), body size 1.30–1.40 mm.
6 (7)	Anterior pronotal angles strongly produced, elytra along widest point rather narrow, ratio width: length 1.14 (Fig. 18), meso-metaventral excavation deep (Fig. 19)	*Sternocoelis marseulii* (Brisout de Barneville, 1866) (Spain)
7 (6)	Anterior pronotal angles weakly produced, elytra along widest point rather broad, ratio width : length 1.20 (Fig. 30), meso-metaventral excavation shallow (Fig. 31)	*Sternocoelis vaucheri* Lewis, 1896 (Morocco: Tangier)
8 (1)	Antennal scape without a ‘hook’	*Sternocoelis viaticus* Lewis, 1892 (Algeria)

#### Sternocoelis yelamosi sp. n.

urn:lsid:zoobank.org:act:6E3C4ACC-F2D0-44F6-8BA0-303324D266A7

http://species-id.net/wiki/Sternocoelis_yelamosi

Figs 1 –16

##### Type locality.

Morocco, Jebel Tazzeka.

##### Type material examined.

Holotype, ♂: MOROCCO: Moyen Atlas / Taza: Jebel Tazzeka / N34°12.226', W04°03.908' / 11.V.2009 / Hlaváč lgt., under stone with ants, 1360 m (printed); // HOST ANT: *Aphaenogaster mauritanicus* Emery / H. Cagniant, det. 2009 (printed); // HOLOTYPE *Sternocoelis yelamosi* Lackner & Hlaváč, det. 2010 (red label, printed); CTLA. **Note.** One specimen of minor worker ant is pinned together with the holotype.

##### Description.

**Figures 1–7.**
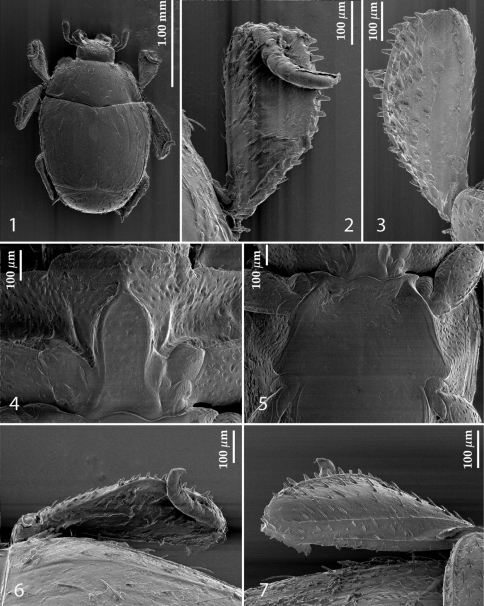
*Sternocoelis yelamosi* sp. n. **1** habitus dorsal view **2** protibia dorsal view **3** ditto, ventral view **4** prosternum **5** meso-metaventrite **6** mesotibia, dorsal view **7** ditto, ventral view.

PEL:1.425; APW:0.625; PPW:1.075; EL:0.875; EW:1.20. Body (Fig. 1) colour reddish-brown, shiny, weakly convex, oval, dorsal surface with sparse setation, ventral surface lacking setae.

Frons wide, almost rugulose-lacunose, coarsely punctate, punctures with sparse setae; eyes well-visible from above; frontal stria carinate, prolonged onto clypeus; antennal scape thickened, apically with a ‘hook’, antennal club cylindrical, truncate at apex.

Pronotum with dense fine punctulation anteriorly and evanescent punctulation laterally and basally, with sparse setae (many setae probably worn off); pronotal sides weakly separated from disc by shallow marginal depression; marginal pronotal stria present on basal three-quarters; anterior pronotal angles produced, truncate; posterior angles of pronotum acute, not produced.

Elytra weakly convex, with long but sparse setae; disc densely punctate, punctures separated by several times their diameter; marginal elytral stria complete, carinate, briefly continued as apical elytral stria; first dorsal elytral stria complete, second dorsal elytral stria reaching approximately two-thirds of elytral length apically, third elytral stria the shortest, fine, reaching approximately mid elytral length apically, other elytral striae absent.

Propygidium about 2.5 times as long as pygidium, both with sparse punctulation and sparse long setae.

Prosternal process (Fig. 4) at slightly higher level than prosternal lobe; carinal prosternal striae bisinuate, joined anteriorly, interspace between them rugose; prosternal lobe roughly punctulate.

Mesoventrite smooth, shiny, asetose; meso-metaventral depression shallow; mesoventral foveae incipient (Fig. 5); three lateral mesoventral striae present; inner one middle of mesoventrite posteriorly. Metaventrite with sparse and fine punctulation present mainly in anterior part, shiny.

All visible abdominal sternites smooth, asetose, second visible abdominal sternite medially about twice as long as sternite I and III.

Legs (Figs 2–3, 6–7) relatively short, with sparse but strong setae, all tibiae expanded from base to apex, with strong setae, mainly on outer margins.

**Figures 8–16.**
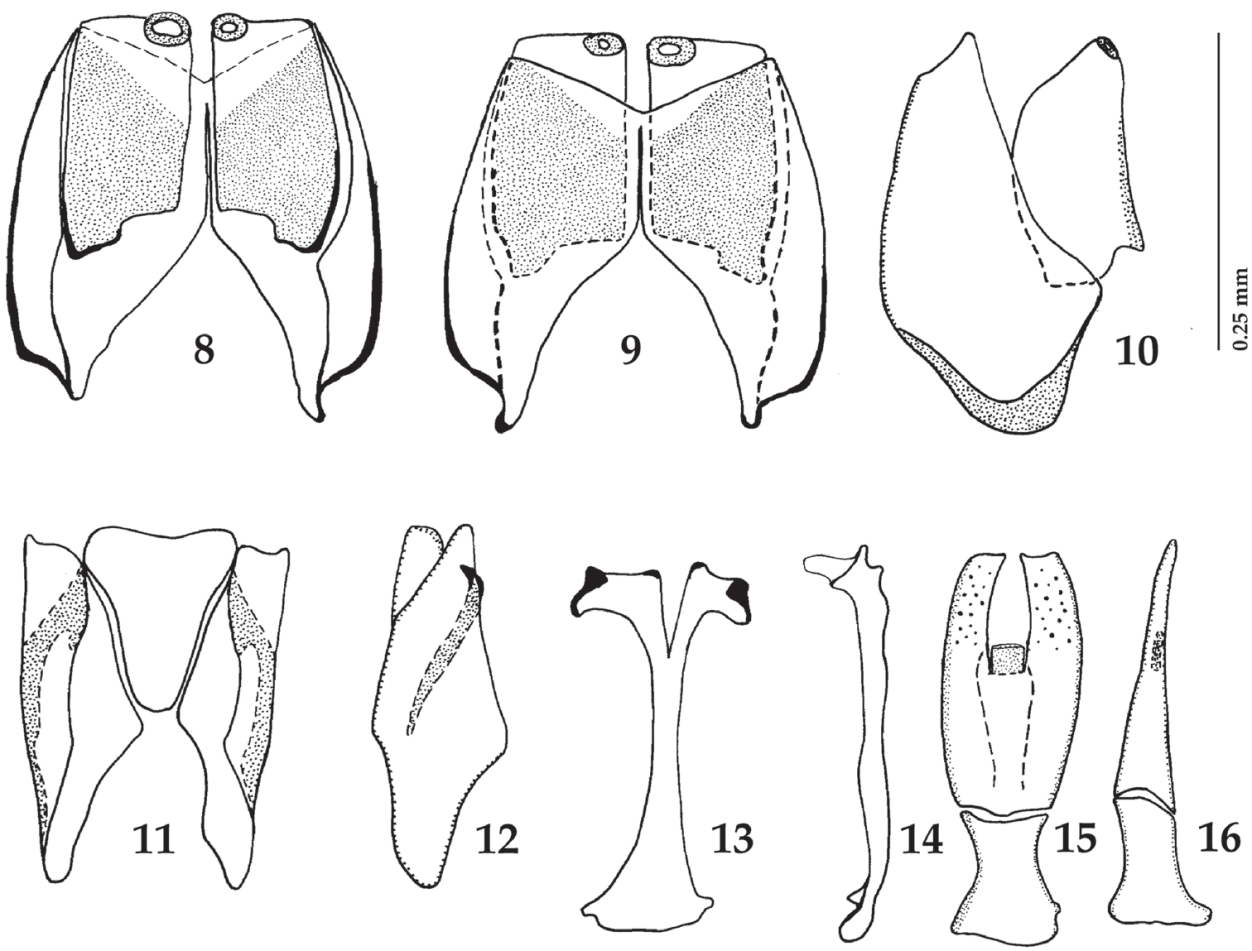
*Sternocoelis yelamosi* sp. n. male terminalia. **8** Eighth sternite and tergite, ventral view **9** ditto, dorsal view **10** ditto, lateral view **11** ninth and tenth tergites, ventral view **12** ditto, lateral view **13** spiculum gastrale, ventral view **14** ditto, lateral view **15** aedeagus, dorsal view **16** ditto, lateral view.

Male genitalia. Eighth sternite (Figs 8–9) longitudinally divided medially, apically without velae, each half of divided eighth sternite apically with round sclerotized ‘ring’; eighth tergite much larger than sternite, longitudinally almost divided medially, its halves widely separated; eighth sternite and tergite completely separated laterally (Fig. 10). Ninth tergite (Figs 11–12) widely separated by tooth-shaped tenth tergite. Spiculum gastrale (Figs 13–14) dilated on both ends, apical end dilated, with medio-apical notch. Aedeagus (Figs 15–16) typical for the genus (see Yélamos 1995: 147, Figs 69–83 for comparison), truncated apically; apical third with pseudopores, laterally barely curved; ratio of basal piece of aedeagus to the parameres approximately 1:2.

##### Differential diagnosis.

*Sternocoelis yelamosi* sp. n. closely resembles other species included in the *Sternocoelis marseulii* species group, but it can be differentiated from all of them as follows: from *Sternocoelis viaticus* by presence of ‘hook’ on antennal scape [lacking only in *Sternocoelis viaticus*]; from *Sternocoelis marseulii* by weaker punctation of the prosternum and coarser punctation of dorsum [prosternum of *Sternocoelis marseulii* is rugose and densely punctate; dorsum with weaker punctation]; from *Sternocoelis vaucheri* by weaker punctation of the prosternum and coarser punctation of elytra [prosternum of *Sternocoelis vaucheri* is rugose, coarsely punctate and elytra are almost impunctate]: from *Sternocoelis berberus* by smaller body size [1.425 *vs*. 1.60 mm in *Sternocoelis berberus*] as well as by weakly explanate pronotal sides [pronotal disc of *Sternocoelis berberus* is clearly separated from strongly explanate pronotal sides].

##### Etymology.

Patronymic, named after and dedicated to our friend Tomás Yélamos (Barcelona, Spain), who revised the genus *Sternocoelis* and confirmed this to be a new species.

##### Distribution.

So far known only from the type locality, Jebel Tazzeka in Middle Atlas, northeast Morocco.

##### Host ant.

*Aphaenogaster mauritanicus* Dalla Torre, 1893.

##### Other material studied.

*Sternocoelis berberus* Lackner &Yélamos, 2001: 26 exs (sex undetermined): Morocco, Oïkameden, 2646m - 2656m (N31°11.605', W07°51.172' – N31°11.609', W07°51.168'), 20.V. 2009, P. Koniar & M. Švarc lgt., under rocks on open slope.

*Sternocoelis viaticus* Lewis, 1892: Paralectotype, ♂, side-mounted on a triangular point with dismembered genitalia glued to the same triangular point as specimen, with another mounting card bearing an ant, with written label: „Meskoutin / G. Lewis / 22.4.[18]92“, followed by another written label „*Sternocoelis* / *viaticus* / Co-Type Lewis“, followed by round, yellow-margined label „Co- / type“ and by another printed label „G. Lewis Coll. / B.M. 1926-369“, followed by another red label, printed „Paralectotypus / T. Yélamos / Des. 1993“.

*Sternocoelis marseulii* (Brisout de Barneville, 1866): 2 ♀♀ & 2 ♂♂ Spain, Escorial (MNHN).

*Sternocoelis vaucheri* Lewis, 1896: Paralectotype, ♂, side-mounted on a triangular point with dismembered genitalia glued to the same triangular point as specimen (spiculum gastrale missing), with another mounting card bearing an ant, with written label: „Tanger / 1896 / Vaucher“, followed by another written label: „Much more / convex than / *Marseulii* Bris.“, followed by printed label: „G. Lewis Coll. / B.M. 1926-369“, followed by another printed label: „*Sternocoelis* / *vaucheri* / Lewis, 1896 / T. Yélamos Det.“, followed by another red label, printed „Paralectotypus / T. Yélamos / Des. 1993“ (BHMN).

## Discussion

*Sternocoelis yelamosi* shares numerous character states with another four species (*Sternocoelis vaucheri*, *Sternocoelis berberus*, *Sternocoelis viaticus* and *Sternocoelis marseulii*) that are found in Morocco, Algeria and Spain, respectively. Most of these character states, according to the phylogenetic analysis performed by Yélamos (1995), are presumed to be symplesiomorphies (Yélamos 1995:168). Yélamos (1995) determined polarities of character states using the Palaearctic genus *Haeterius* Dejean, 1833 for the out-group. Yélamos (1995) did not study male terminalia in detail; he only mentioned that “...male genitalia are very constant, with an almost imperceptible intraspecific variability” (Yélamos 1995: 168). According to our study of the male terminalia of members of the ‘*Sternocoelis marseulii* species group’ the most reliable character for distinguishing species of this group is the shape of spiculum gastrale, especially its apical and basal ends (compare Figs 13, 26, 37 and 45). *Sternocoelis marseulii* is the only species of *Sternocoelis marseulii* species group that already had its spiculum gastrale illustrated (Yélamos 1995: 147, Fig. 84). However, our study of the male terminalia of *Sternocoelis marseulii* indicates that the spiculum gastrale of this species (Fig. 26) is rather different from that illustrated by Yélamos (1995). The rest of the male terminalia (Figs 8–12, 14–16; 21–25, 27–29; 32–36, 38–39; 40–44, 46–48) show more uniformity and are less valuable tools for the intra-specific recognition. The spiculum gastrale of *Sternocoelis vaucheri* could not have been examined; therefore the validity of this taxon remains dubious. *Sternocoelis vaucheri* is known only from two males and two specimens of unidentified sex, all collected more than hundred years ago in Tangier (northern Morocco). Two males of this species belonging to the type series housed in BMNH have been examined, unfortunately their male terminalia (except for the aedeagus already drawn by Yélamos 1995) are damaged and unsuitable for drawing. According to Yélamos (1995), there should be another two specimens of unidentified sex housed in MNHN; these two specimens, however, have not been found in the collections (Taghavian pers. comm., 2011). A lot of effort was devoted to find this rare species in the surroundings of Tangier, Morocco, but without success. According to Yélamos (pers. comm. 2011), *Sternocoelis vaucheri* might be a junior synonymy of *Sternocoelis marseulii*. *Sternocoelis marseulii* occurs predominantly in the mountains of central and eastern Spain, with few findings also in southern Spain (Yélamos 1995). One locality, Sierra de Córdoba is approximately 300 km from the type locality of *Sternocoelis vaucheri* (Tangier, Morocco). However, without the examination of the male terminalia of *Sternocoelis vaucheri* it would be premature to synonymize the two species and therefore both species are kept in their current taxonomic status. We believe that a newly performed phylogenetic analysis including the male terminalia, with desired molecular characters would help to elucidate the relationships among the *Sternocoelis* species.

**Figures 17–20.**
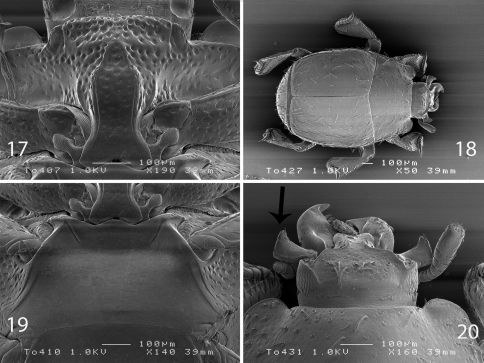
*Sternocoelis marseulii* (Brisout de Barneville, 1866). **17** prosternum **18** habitus, dorsal view **19** meso-metaventrite **20** head, dorsal view.

**Figures 21–29.**
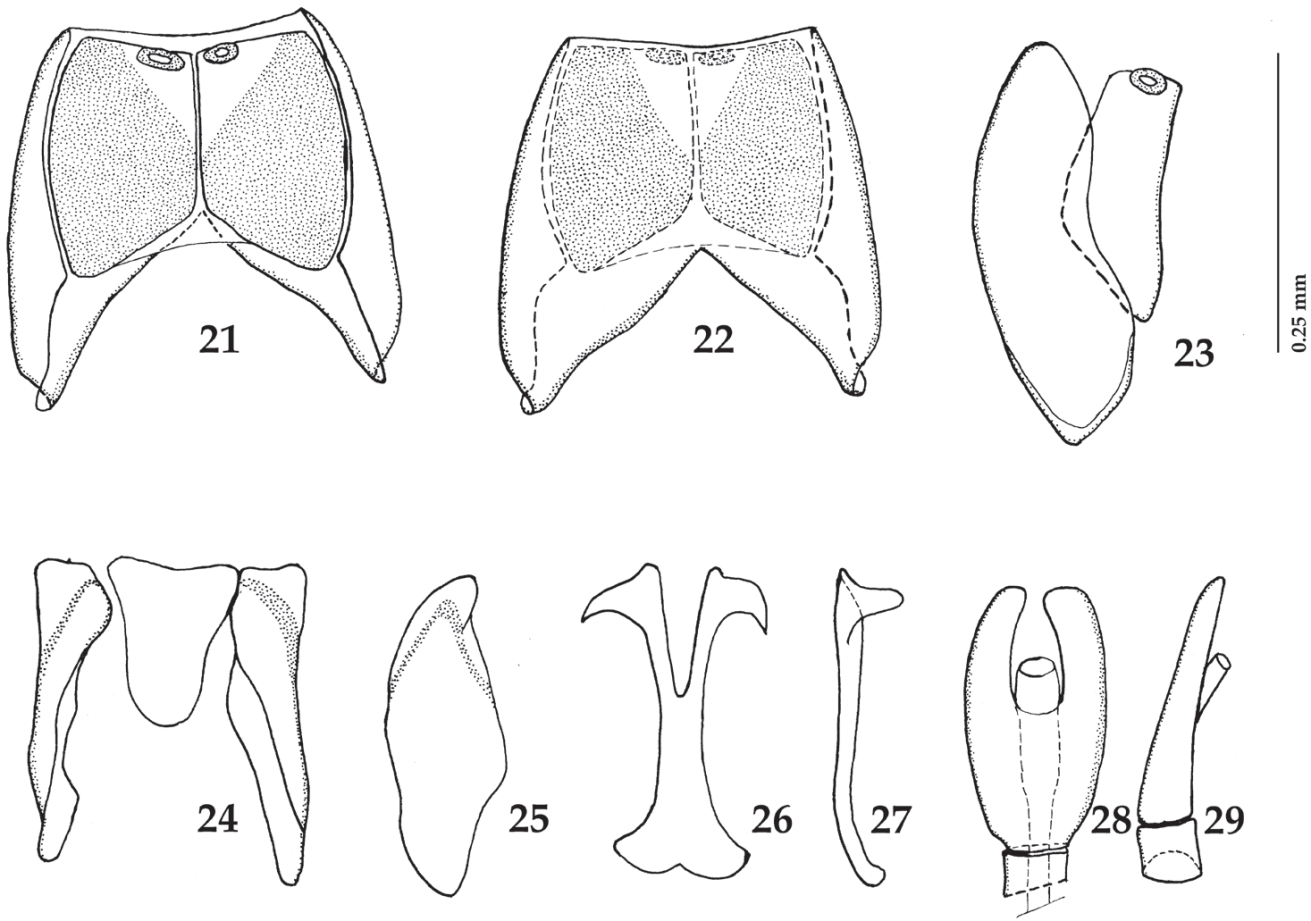
*Sternocoelis marseulii* (Brisout de Barneville, 1866) male terminalia. **21** Eighth sternite and tergite, ventral view **22** ditto, dorsal view **23** ditto, lateral view **24** ninth and tenth tergites, ventral view **25** ditto, lateral view **26** spiculum gastrale, ventral view **27** ditto, lateral view **28** aedeagus, dorsal view **29** ditto, lateral view.

**Figures 30–31.**
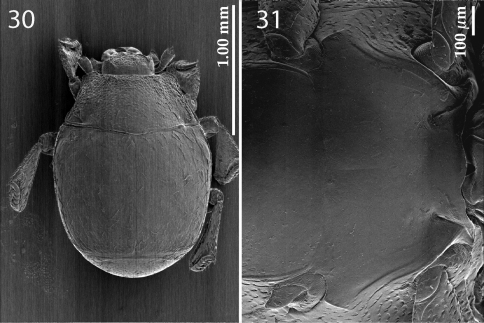
*Sternocoelis vaucheri* Lewis, 1896. **30** habitus, dorsal view **31** meso-metaventrite.

**Figures 32–39.**
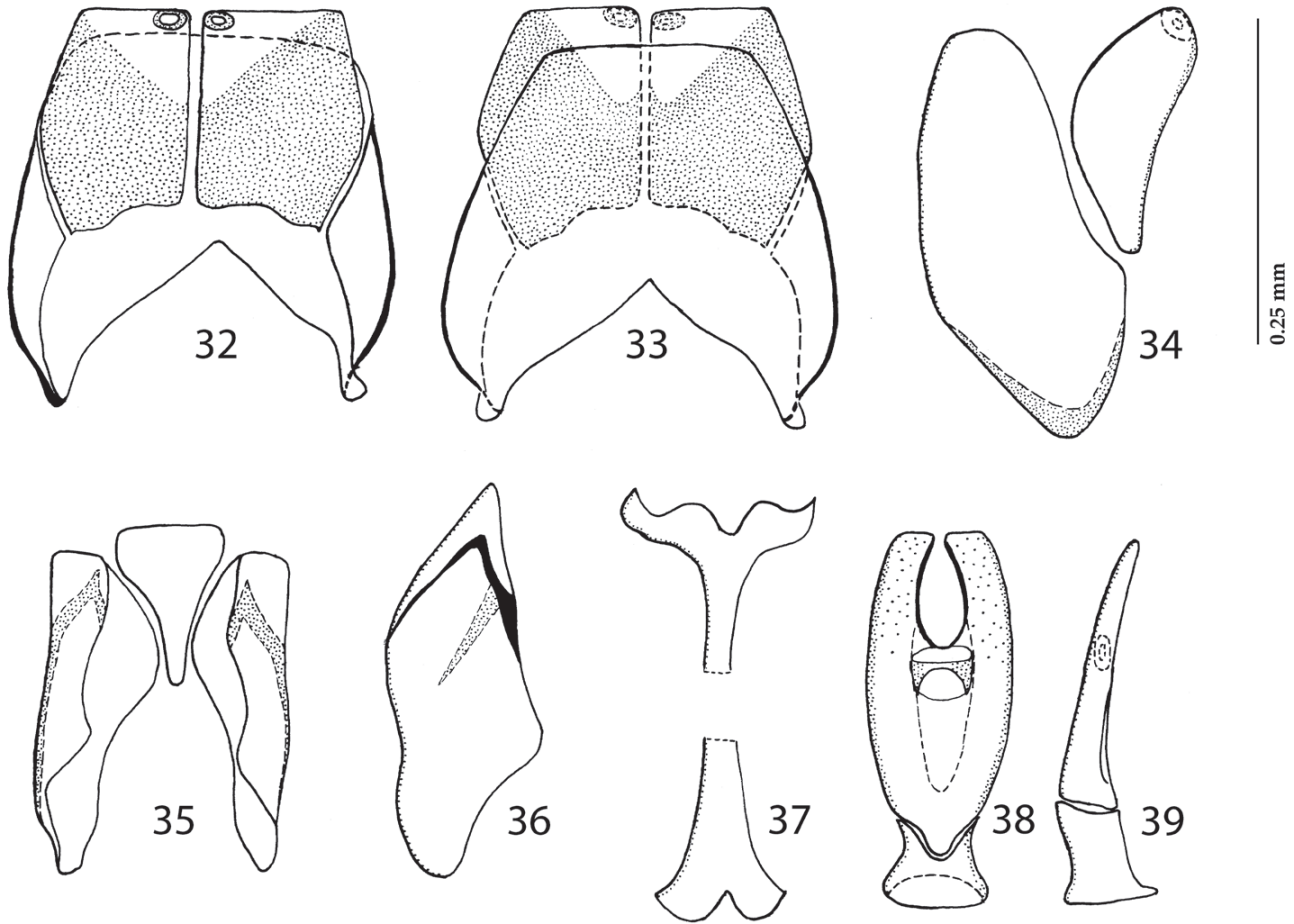
*Sternocoelis berberus* Lackner & Yélamos, 2001, male terminalia. **32** Eighth sternite and tergite, ventral view **33** ditto, dorsal view **34** ditto, lateral view **35** ninth and tenth tergites, ventral view **36** ditto, lateral view **37** spiculum gastrale, ventral view **38** aedeagus, dorsal view **39** ditto, lateral view.

**Figures 40–48.**
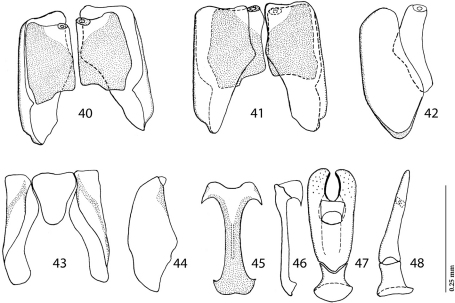
*Sternocoelis viaticus* Lewis, 1892, male terminalia. **40** Eighth sternite and tergite, ventral view **41** ditto, dorsal view **42** ditto, lateral view **43** ninth and tenth tergites, ventral view **44** ditto, lateral view **45** spiculum gastrale, ventral view **46** ditto, lateral view **47** aedeagus, dorsal view **48** ditto, lateral view.

## Supplementary Material

XML Treatment for Sternocoelis yelamosi

## References

[B1] Lackner T (2010) Review of the Palaearctic genera of Saprininae. Acta Entomologica Musei Nationalis Pragae, Supplement 50: 1-254

[B2] Lackner T Yélamos T (2001) Contribution to the knowledge of the Moroccan fauna of *Sternocoelis* Lewis, 1888 and *Eretmotus* Lacordaire, 1854 (Coleoptera: Histeridae). ZAPATERI, Revista Aragonesa de Entomologia 9: 99-102

[B3] Mazur S (1997) A world catalogue of the Histeridae (Coleoptera: Histeridae). Genus, Supplement, 373 pp.

[B4] Ôhara M (1994) A revision of the superfamily Histeroidea of Japan (Coleoptera). Insecta Matsumurana (N. S.) 51: 1-238

[B5] Yélamos T (1995) Revision of the genus *Sternocoelis* Lewis, 1888 (Coleoptera: Histeridae), with a proposed phylogeny. Revue Suisse de Zoologie 102 (1): 113-174

